# Three-dimensional-printed custom-made hemipelvic endoprosthesis for primary malignancies involving acetabulum: the design solution and surgical techniques

**DOI:** 10.1186/s13018-019-1455-8

**Published:** 2019-11-27

**Authors:** Jie Wang, Li Min, Minxun Lu, Yuqi Zhang, Yitian Wang, Yi Luo, Yong Zhou, Hong Duan, Chongqi Tu

**Affiliations:** 1 Department of Orthopedics, West China Hospital, Sichuan University, No. 37 Guoxuexiang, Chengdu, 610041 Sichuan People’s Republic of China; 2 Bone and Joint 3D-Printing & Biomechanical Laboratory, Department of Orthopedics, West China Hospital, Sichuan University, No. 37 Guoxuexiang, Chengdu, 610041 Sichuan People’s Republic of China

**Keywords:** 3D-printed, Endoprosthesis, Hemipelvic reconstruction, Design solution, Surgical technique

## Abstract

**Background:**

This study is to describe the detailed design and surgical techniques of three-dimensional (3D)-printed custom-made endoprosthesis for hemipelvic tumorous bone defect.

**Methods:**

According to the pelvic tumor resection classification by Enneking and Dunham, the hemipelvis is divided into three zones including the ilium (P1), acetabulum (P2), and pubis and ischium (P3). Thirteen patients were included in this study. Of these, P1 and P2 were involved in three cases, while P1, P2, and P3 were involved in 10. Based on radiography data, 3D pelvic model was rebuilt, and virtual surgery was simulated. Different fixation methods were applied according to residual bone volume. Parameters of the first sacral (S_1_) vestibule, second sacral (S_2_) vestibule, the narrowest zone of superior pubic medullary cavity (NPSPMC), and the resected surface of superior pubic medullary cavity (RSSPMC) were selectively measured in various fixation methods. Model overlapping, feature simplifying, and size controlling were three basic steps during design procedure. Volume proportion of porous structure was determined according to estimated weight of resected specimen. Acetabular location, anteversion, and inclination were modulated. Screw diameter, direction, and combination were considered. The osteotomy guides and plastic models were used during surgery.

**Results:**

Of 13 cases, after P1 resection, endoprostheses were fixed to sacra (8; 61.5%), ilia (3; 23.1%), and both (2; 15.4%). After P3 resection, endoprostheses were fixed to residual acetabulum (3; 23.1%), and residual pubis by stem (8; 61.5%) or “cap-like” structure (2; 15.4%). Mean area of the S_1_ vestibule, S_2_ vestibule, RSSPMC, and PSPMC were 327.9 (222.2 to 400), 131.7 (102.6 to 163.6), 200.5 (103.8 to 333.2), and 79.8 mm^2^ (40.4 to 126.2), respectively. Porous structure with 600 μm pore size and 70% porosity accounted for 68.8% (53.0 to 86.0) of the whole endoprosthesis on average. Mean acetabular anteversion and inclination were designed as 23.2° (20 to 25) and 42.4° (40 to 45). Median numbers of screws designed in the S_1_ vestibule was 5 (IQR, 4 to 6), in the S2 vestibule was 1 (IQR, 1 to 2), in the ilium was 5 (IQR, 2 to 6), and in the pubis was 1 (IQR, 1 to 1), while screws designed in the ischium was all 2. Median number of screws inserted in the S_1_ vestibule was 4 (IQR, 3 to 4), in the S_2_ vestibule was 1 (IQR, 1 to 1), in the ilium was 3 (IQR, 1 to 5), in the pubis was 1 (IQR, 0 to 1), and in the ischium was 1 (IQR, 1 to 1).

**Conclusions:**

This study firstly presents detailed design and related surgical techniques of 3D-printed custom-made hemipelvic endoprosthesis reconstruction. Osseointegration is critical for long-term outcome and requires three design elements including interface connection, porous structure, and initial stability achieved by precise matching and proper fixation methods.

## Background

Of all primary malignancies, 5–15% sarcomas occur in the pelvis, and chondrosarcoma, and Ewing's sarcoma along with osteosarcoma are the top three tumors [1, 2]. Besides, pelvis is reported as the third most common site of bone metastases following the spine and ribs [3]. Among pelvic tumors, tumors involving the acetabulum are closely related to poor hip joint function; therefore, how to treat them deserves special attention [4, 5].

Currently, en-bloc resection has been widely accepted, but correct and precise reconstruction of hemipelvic bone defect with low complication rate remains challenging. Previously, numerous reconstruction methods have been proposed, including iliofemoral arthrodesis or pseudarthrosis [6], massive allograft [7], and autoclaved autograft [8]; however, a large amount of studies revealed various limitations of these reconstruction options, such as highly occurred complications or poor functional results. In recent years, prosthetic reconstruction has predominated, on account of its easier approachability, better initial stability, more acceptable cosmesis, earlier weight bearing, and relatively rapid restoration of function [2].

In order to improve the implantation convenience and fixation stability, a lot of prostheses with different shapes and fixation methods has been reported, such as saddle endoprosthesis [1, 9], ice cream cone endoprosthesis [10, 11], modular hemipelvic endoprosthesis [2], and custom-made hemipelvic endoprosthesis [12–14]. Nevertheless, bone implant integration is still unsatisfying in long-term follow-up due to outdated interfacial processing of the endoprosthesis and poor matching between endoprosthesis and residual host bone. To improve long-term survival of endoprosthesis, advanced technique for anatomy-imitating prostheses with a more efficiently osteoconductive structure is urgently demanded.

With understanding and progress on material science and three-dimensional (3D) printing technology (additive manufacturing), 3D-printed custom-made endoprosthesis with porous structure is considered to be a solution for these complicated and irregular bone defects, such as pelvic tumorous defect. To our best knowledge, 3D-printed custom-made hemipelvic endoprosthesis has already been reported with a promising result [15]. But previous reports mostly focused on clinical applications, the detailed design principles and methodology of this kind of endoprosthesis has not been described. Additionally, the worldwide criteria for 3D-printed endoprosthesis is still unestablished. Therefore, research and discussion on the design and methodology of 3D-printed endoprosthesis are inevitable.

Recently, we designed a kind of 3D-printed custom-made hemipelvic endoprosthesis and applied them in treating patients. Favorable clinical outcomes were observed. In this study, our aim is to describe the detailed design and surgical techniques of this endoprosthesis for hemipelvic bone defect.

## Methods

### Patients

Between 2016 and 2017, 13 patients with malignant tumors involving the acetabulum received hemipelvic replacement with 3D-printed custom-made endoprosthesis. There were six males and seven females with a mean age of 48.7 years (31 to 66). The three main areas of the pelvis where tumor arises have been classified by Enneking and Dunham as the ilium (P1), acetabulum (P2), and pubis and ischium (P3) [16]. Totally, P1 and P2 were involved in three cases, while P1, P2, and P3 were involved in 10. According to the Enneking staging system [17], ten patients with chondrosarcoma, fibrosarcoma, solitary plasmacytoma, and angiosarcoma were stage IIB; three patients with parosteal osteosarcoma, Ewing's sarcoma, and osteosarcoma were stage III (Table 1). All patients underwent plain radiography (PR), 3D computerized tomography (3D-CT), and magnetic resonance imaging (MRI) of lesions. Single-photon emission computed tomography (SPECT) or positron emission tomography/ computerized tomography (PET/CT) and biopsy were also performed preoperatively.

**Table 1:** The demographics of the 13 patients treated with 3D-printed custom-made hemipelvic endoprosthesis

Case	Age (years)	Gender	Resection classification^a^	Diagnosis	Enneking staging	Follow-up (months)
1	46	F	P1+P2+P3	Parosteal osteosarcoma	III	29.0
2	37	F	P1+P2+P3	Chondrosarcoma	IIB	28.0
3	48	M	P1+P2+P3	Chondrosarcoma	IIB	27.0
4	65	F	P1+P2+P3	Fibrosarcoma	IIB	23.0
5	31	F	P1+P2+P3	Ewing’s sarcoma	III	27.0
6	61	M	P1+P2	Solitary plasmacytoma	IIB	26.0
7	40	M	P1+P2	Chondrosarcoma	IIB	25.0
8	40	M	P1+P2+P3	Chondrosarcoma	IIB	25.0
9	46	F	P1+P2	Chondrosarcoma	IIB	24.0
10	66	M	P1+P2+P3	Fibrosarcoma	IIB	22.0
11	53	F	P1+P2+P3	Osteosarcoma	III	26.0
12	35	M	P1+P2+P3	Angiosarcoma	IIB	24.0
13	65	F	P1+P2+P3	Chondrosarcoma	IIB	23.0
Mean	48.7	-	-	-	-	25.3

^a^According to Enneking and Dunham [16]

### Anatomical measurement

**Fig. 1:**
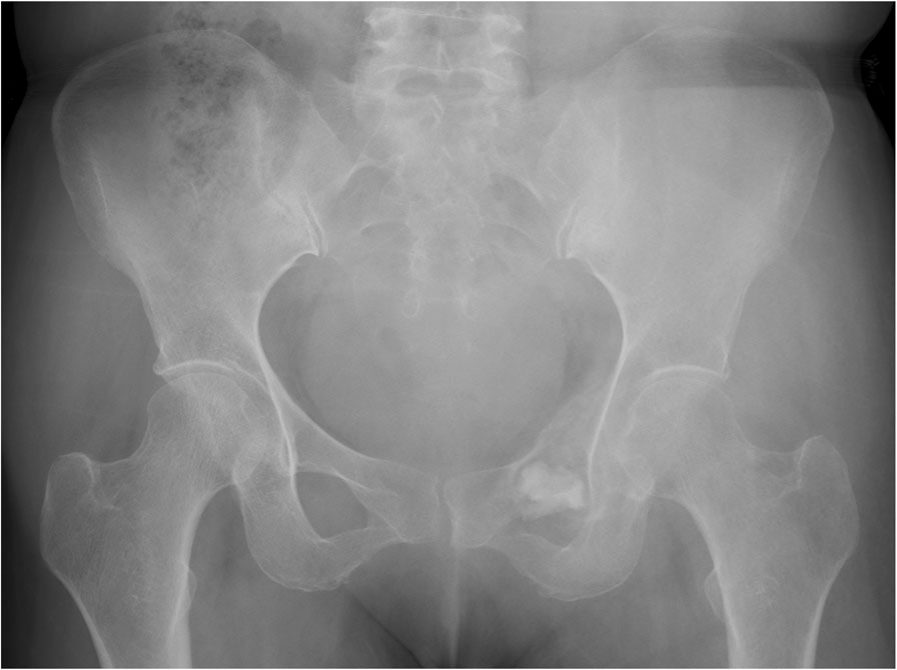
Pelvic plain radiography in a patient with an osteosarcoma (after biopsy) involving the anterior column of left acetabulum, ischium, pubis, and ilium

**Fig. 2:**
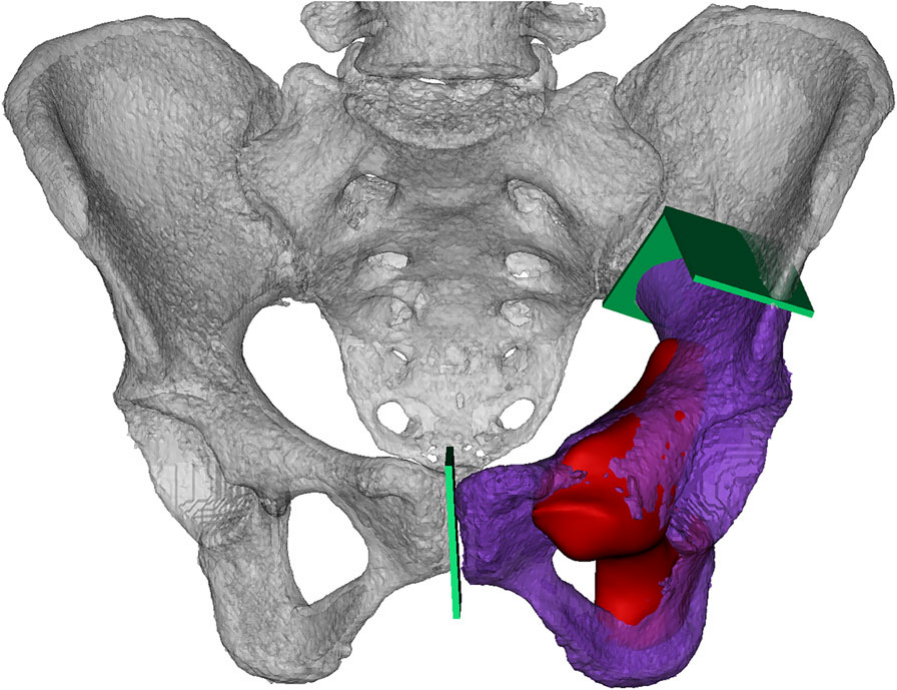
Rebuilding pelvis (white) and tumor model (red) with CT and MRI, and simulating en-bloc resection. The resected specimen (purple) and osteotomy plain (green) were exhibited

Virtual 3D pelvic model was built by importing 3D-CT scanning data into Mimics V20.0 software (Materialise Corp., Leuven, Belgium). Information about tumor size and extension provided by MRI was integrated into a virtual 3D pelvic model to show tumor margin visually. The tumor size was measured in length, width, and height (Table 2). En-bloc resection was simulated (Figs.1 and 2). Resection margin was set as 10 mm for chondrosarcoma and 30 mm for high-grade malignancies such as osteosarcoma and Ewing's sarcoma. Target host structures for endoprosthesis fixation were analyzed and measured.

**Table 2:** Detailed information of measured data

Case	Size of S_1_ vestibule			Size of S_2_ vestibule			Size of NPSPMC			Size of RSSPMC			Anatomical AO		Tumor size
	A(mm^2^)	L(mm)	W(mm)	A(mm^2^)	L(mm)	W(mm)	A(mm^2^)	L(mm)	W(mm)	A(mm^2^)	L(mm)	W(mm)	An (°)	I (°)	(cm^3^)
1	334.9	22.3	20.2	118.2	15.1	9.3	107.8	13.8	10.0	310.1	41.1	9.6	21.0	44.5	12×18×13
2	222.2	19.5	15.8	102.6	13.9	8.9	40.4	8.6	6.2	146.2	24.6	7.6	27.2	44.4	11×16×10
3	335.1	22.4	19.7	121.7	15.1	11.4	61.9	10.4	7.0	333.2	34.4	12.4	18.9	38.4	10×15×14
4	330.8	24.5	16.3	142.1	16.5	12.1	47.7	9.2	6.9	105.9	22.5	5.7	20.0	41.3	7×12×12
5	286.8	22.7	16.5	138.1	15.7	11.7	76.2	13.0	7.0	118.0	19.9	8.2	23.1	42.4	14×16×13
6	316.0	22.1	18.1	107.1	15.5	7.8	66.9	11.0	7.8	-	-	-	18.6	43.2	5×6×4
7	400.0	27.0	19.4	163.6	17.3	12.0	87.3	12.0	9.3	103.8	14.2	9.3	18.8	45.8	7×11×7
8	-	-	-	-	-	-	86.0	12.5	8.1	172.1	19.5	10.9	18.3	40.1	4×7×4
9	-	-	-	-	-	-	74.1	12.1	8.4	-	-	-	21.5	38.8	6×6×4
10	344.6	22.1	21.0	130.6	15.7	10.0	126.2	16.3	9.9	281.4	38.0	7.7	18.9	41.2	7×12×9
11	-	-	-	-	-	-	77.0	11.5	8.5	217.1	30.2	9.2	21.8	39.9	5×7×8
12	383.0	25.5	19.3	147.6	16.6	10.8	89.5	13.8	8.3	-	-	-	15.0	40.5	5×6×6
13	316.0	23.3	17.5	145.4	15.7	11.7	96.2	12.0	10.0	217.5	29.6	7.6	19.3	41.6	7×10×7
Mean	327.9	23.1	18.4	131.7	15.7	10.6	79.8	12.0	8.3	200.5	27.4	8.8	20.2	41.7	7.7×10.9×8.5

S_1_, first sacral; S_2_, second sacral; NPSPMC, the narrowest part of superior pubic medullary cavity; RSSPMC, the resected surface of superior pubic medullary cavity; A, area; L, length; W, width; AO, acetabular orientation; An, anteversion; I, inclination

After P1 resection, measurement was alternative depending on whether the sacroiliac joint was involved or not. If the sacroiliac joint was involved, sizes of the first sacral (S_1_) and second sacral (S_2_) vestibules, the narrowest part of the bony corridor from the lateral ilium to the sacral body would be measured [18]. If not, no measurement would be performed.

Anatomical acetabular anteversion and inclination were measured in all cases.

**Fig. 3:**
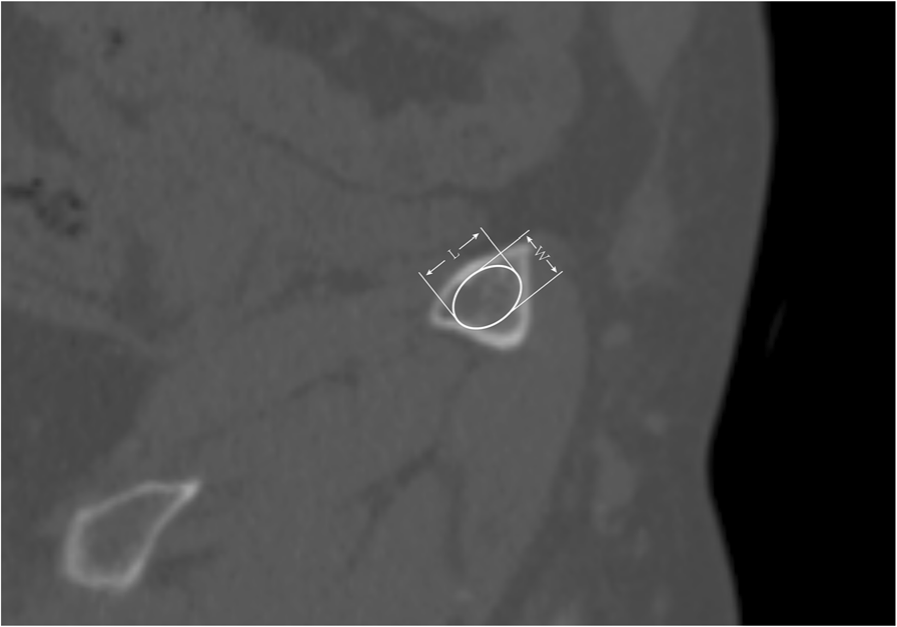
Length (L) and width (W) measurement at pubic ramus, two cross-section surfaces might be measured including the resected surface and the narrowest part

After P3 resection, the size of the narrowest zone of superior pubic medullary cavity (NPSPMC) was all measured (Fig. 3), and the size of the resected surface of superior pubic medullary cavity (RSSPMC) was measured depending on whether the pubis was involved or not. If the pubis was involved, the size of RSSPMC was measured directly in cases whose pubic ramus was partially resected, while in cases resected from pubic symphysis, the size of the medullary cavity in sagittal plane near the pubic symphysis was measured. If not, no more measurement would be performed. No measurement related to the ischium was performed.

### Endoprosthesis design and fabrication

All endoprostheses were designed by our clinical team and fabricated by Chunli Co., Ltd., Tongzhou, Beijing, China. The preliminary shape of endoprosthesis was generated from bone defect after virtual resection by overlapping the mirror model at the corresponding region of the contralateral pelvis. The endoprosthesis feature and size was modified. The endoprosthesis was composed of solid and porous structures. Endoprosthesis weight was modulated by adjusting the volume proportion of the porous structure (VPPS). After calculating the mean density of porous structure and measuring the volume of resected specimen and total endoprosthesis in Mimics, VPPS was calculated according to the following formula:

$$ V\mathrm{r}\times \frac{\rho \mathrm{tmin}+\rho \mathrm{tmax}}{2}=\rho \mathrm{Ti}\times V\mathrm{e}\times \left(1-\mathrm{VPPS}\right)+\rho \mathrm{P}\times V\mathrm{e}\times \mathrm{VPPS} $$

$$ \mathrm{VPPS}=1.095-0.13\frac{V\mathrm{r}}{V\mathrm{e}} $$

Where the *ρ*_tmin_ and *ρ*_tmax_ are the minimum and maximum density of pelvic trabecular bone, which are 0.109 and 0.959 g/ml, respectively [19], *ρ*_Ti_ is the density of titanium alloy, 4.51 g/ml, *ρ*_P_ is the mean density of porous structure, 0.39 g/ml, *V*e is the volume of total endoprosthesis, and *V*r is the volume of resected specimen.

**Fig. 4:**
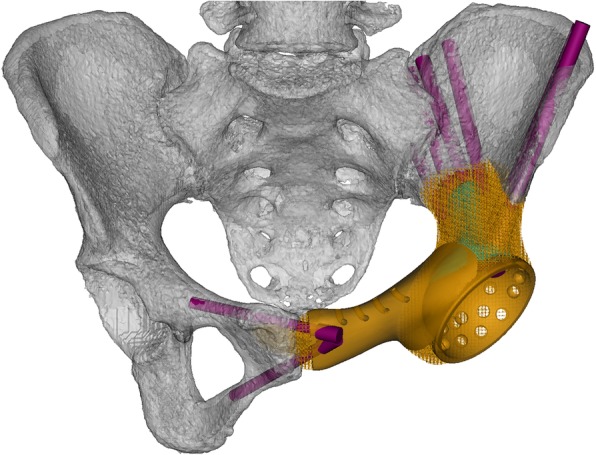
The endoprosthesis (orange) was composed of solid structure and porous structure, and matched bone defect. “Arc-like” supporting structure (cyan) distributed along arcuate line. Six radial screws fixed the endoprosthesis to the residual ilium, and two screws fixed the endoprosthesis to contralateral pubis

Porous structures were generated in Magics V22.0 software (Materialise Corp., Leuven, Belgium). Acetabular location and orientation, including anteversion and inclination, were modulated in Mimics. Both after P1 and P3 resections, fixation methods were determined by residual bone volume in that region. And better initial stability of endoprosthesis was achieved by inserting more and longer screws, which was according to sizes of the S_1_ vestibule, S_2_ vestibule, and NPSPMC (Fig. 4). The osteotomy guides were generated by Boolean Operations of the models. Uninvolved bone surface for seating osteotomy guides were confirmed by MRI. The solid plastic endoprosthesis model was created without pubic stem.

**Fig. 5:**
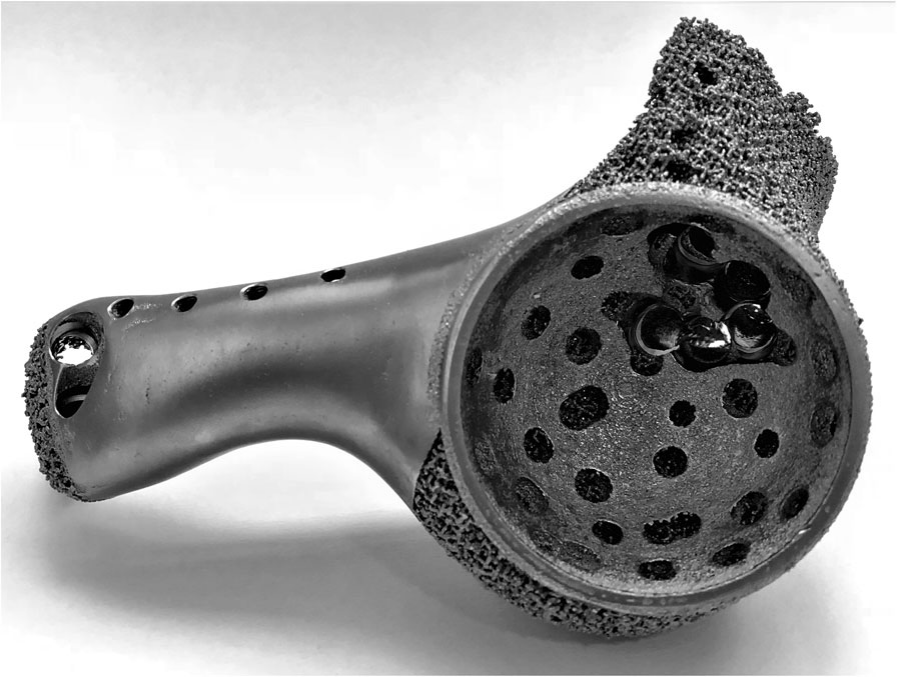
Anterior view of the endoprosthesis fabricated by electron beam melting technique. The porosity was 70% and pore size was 600 μm

**Fig. 6:**
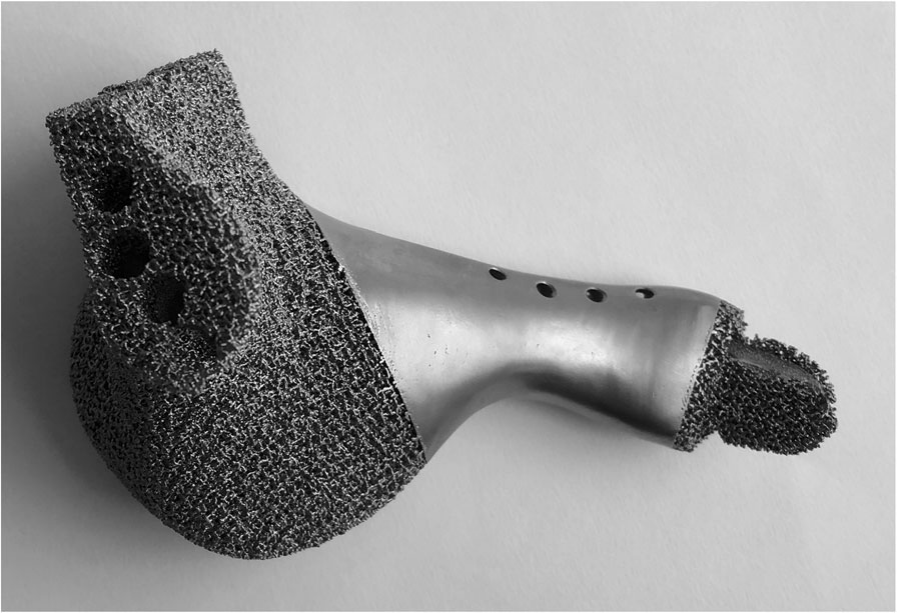
Posterior view of the endoprosthesis fabricated by electron beam melting technique. The porosity was 70% and pore size was 600 μm

The endoprosthesis was fabricated by electron beam melting technique (ARCAM Q10plus, Mölndal, Sweden, Figs. 5 and 6). Meanwhile, the osteotomy guides and plastic endoprosthesis models were fabricated by stereo lithography appearance technique (UnionTech Lite 450HD, Shanghai, China). The endoprosthesis was weighed with a digital scale with an accuracy of 0.5 g before sterilized. All the design procedures took two days. Three-dimensional printing fabrication, post-processing, and delivery cost took three, two, and three days, respectively.

### Surgical techniques

**Fig. 7:**
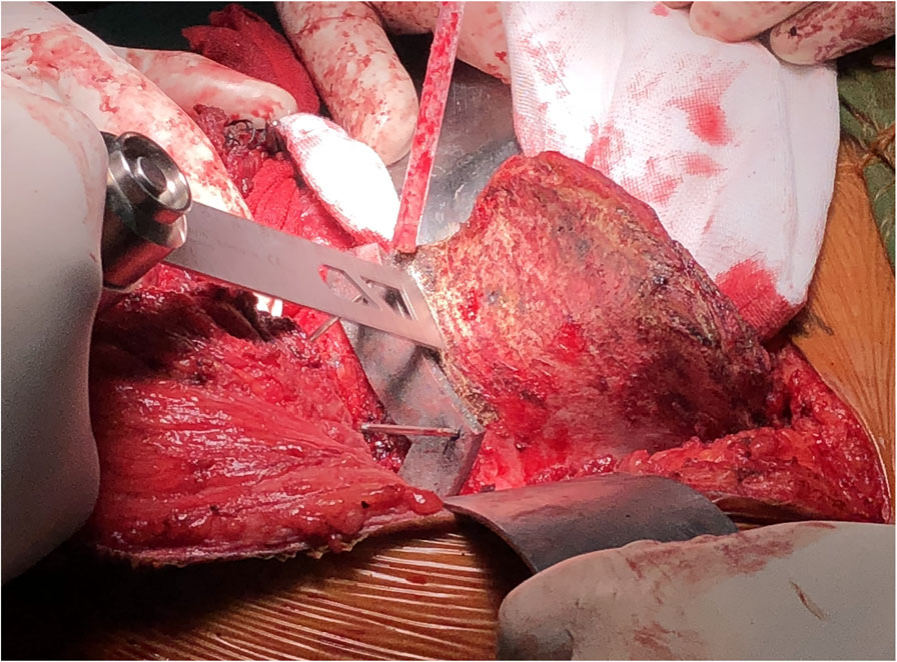
Resection with the aid of osteotomy guide. The osteotomy guide hooked the greater sciatic notch and anterior inferior iliac spine, then stabilized by three 1.8 mm-diameter Kirschner's wires

**Fig. 8:**
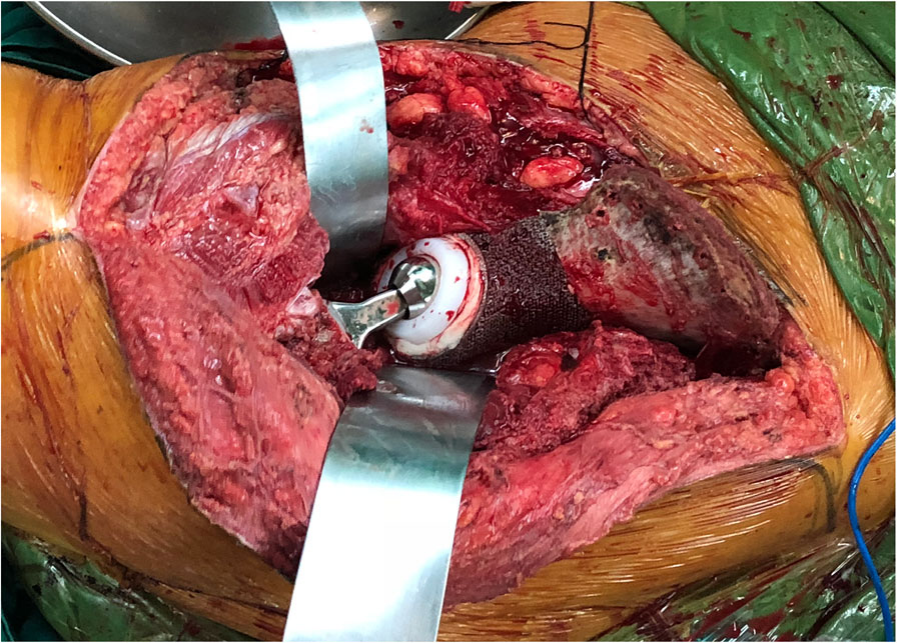
The perfect match was achieved, and residual iliac bone was drilled to improve local circulation

All surgeries were performed by the same senior surgeon. Lateral position and combination of a posterior iliac and Smith-Petersen approach, with or without ilioinguinal approach, were applied. Soft tissue was removed to expose sufficient bone surface for seating osteotomy guides. Osteotomy was performed after stabilizing the osteotomy guides by Kirschner wires (Fig. 7). The plastic implant trial was used to confirm perfect fit before the definitive endoprosthesis was inserted. Sacral or iliac fixation was done in priority. After prosthetic fixation to the sacrum or ilium, reduction of the entire pelvis was conducted and endoprosthesis was fixed to the residual pelvis by screw inserting to the pubic ramus. The ischium was the last one to fix if it was preserved. After establishing rigid fixation, an acetabular component was cemented into the prosthetic acetabulum and total hip arthroplasty thereafter (Fig. 8). Intraoperative time and blood loss were recorded.

### Postoperative management

All patients underwent postoperative pelvic PR and CT to assess acetabular orientation. Further evaluations including physical examination, PR, and tomosynthesis-Shimadzu metal artifact reduction technology (T-SMART) of the pelvis were performed monthly in the first three months and trimonthly thenceforward. Osseointegration was evaluated by T-SMART. Functional outcome was assessed with Musculoskeletal Tumor Society (MSTS) score at latest follow-up [20]. The MSTS score measures patient activity, including pain, function, emotional acceptance, supports, walking ability, and gait. Each variable was assessed on a 5-point scale. Range of motion (ROM) of hip joint was measured. Complications were recorded. Resection margin was assessed and reported by the pathology department of our institution.

### Statistical analysis

Statistical analyses were performed using IBM SPSS Statistics software, version 25 (IBM SPSS, Armonk, NY). Descriptive statistics including proportion and mean value were calculated. Anatomical, designed, and postoperative orientation of the acetabulum were compared using paired-*t* test. A *p* < 0.05 was considered statistically significant.

## Results

Totally, 13 endoprostheses were designed on the basis of measured data. The mean tumor size was 7.7 (4 to 14) × 10.9 (6 to 18) × 8.5 (4 to 14) cm^3^ (Table 2).

As we have mentioned above, the endoprosthesis was composed of solid and porous structures. A 20-mm wide,and 5-mm thick solid titanium “arc-like” supporting structure was distributed from osteotomy plane after P1 resection, extended along arcuate line and ended at osteotomy plane after P2/P3 resection, connecting the other solid structures such as the screw holes, acetabulum, and pubis (Fig. 4). The porous structure was 600 μm pore size and 70% porosity. Averaged estimated resected volume endoprosthesis volume were 488.0 (186.7 to 732.8) and 155.9 ml (75.6 to 266.1). Averaged VPPS was 68.8% (53.0 to 86.0%), and mean endoprosthesis weight was 256.4 g (109.4 to 394.5). In cases whose iliac crest was reconstructed, small holes were distributed along the crest (Tables 2 and 3).

**Table 3:** Detailed data of endoprosthesis design

Case	Sacroiliac joint		Pubic ramus		Ischium		Designed AO		*V*_r_	*V*_e_	*W*_e_ (g)	VPPS
	involvement		RS	RC	RS	RC	A (°)	I (°)	(ml)	(ml)		(%)
1	N		Total	Stem	Total	N	20	43	732.8	266.1	394.5	73.7
2	Y		Total	Stem	Total	N	23	43	543.8	137.8	289.5	58.2
3	Y		Total	Stem	Total	N	23	43	709.7	163.3	377.0	53.0
4	Y		Partial	Cap-like structure	Total	N	24	42	700.6	212.8	374.9	66.7
5	Y		Partial	Stem	Partial	Y	25	41	510.6	190.2	275.1	74.6
6	Y		N	N	N	N	25	40	186.7	96.7	102.1	84.4
7	Y		Partial	Stem	Partial	Y	23	42	697.2	164.2	370.5	54.3
8	N		Partial	Cap-like structure	Partial	Y	23	45	494.8	128.4	263.6	59.4
9	N		N	N	N	N	24	42	202.9	75.6	109.4	74.6
10	Y		Partial	Stem	Partial	Y	24	40	474.2	204.8	189.9	79.4
11	N		Total	Stem	Total	N	20	45	412.3	98.7	219.2	55.2
12	Y		N	N	N	N	25	40	203.2	112.4	111.5	86.0
13	Y		Partial	Stem	Partial	Y	22	42	475.7	175.7	256.2	74.3
Mean	-	-	-	-	-	-	23.2	42.4	488.0	155.9	256.4	68.8

RS, resection; RC, reconstruction; Y, yes; N, no; AO, acetabular orientation; A, anteversion; I, inclination; *V*_r_, volume of resect specimen; *V*_e_, volume of endoprosthesis; *W*_e_, weight of endoprosthesis; VPPS, volume proportion of porous structure.

After P1 resection, fixation methods were determined by the volume of the residual ilium. Total resection of the ilium was performed in eight cases, endoprosthesis was directly fixed to the sacrum with a flat connecting surface. Orientation and number of ilio-sacral screws were designed according to the size of S_1_ and S_2_ vestibules. In three cases, most of the ilium was preserved, and endoprosthesis was fixed only to the ilium. Number and orientation of screws fixing in the iliac crest were modulated. Additionally, in two cases, most of the ilium was resected, the endoprosthesis was fixed to the sacrum by the same way as that after total resection of the ilium and to the residual ilium by screws (Table 3).

Therefore, in our series, prostheses were fixed to the sacrum in 10 cases. Mean area, length, and width of S_1_ vestibule were 327.9 mm^2^ (222.2 to 400.0), 23.1 mm (19.5 to 27), and 18.4 mm (15.8 to 21.0), respectively. The average area, length, and width of the S_2_ vestibule were 131.7 mm^2^ (102.6 to 147.6), 15.7 mm (13.9 to 17.3), and 10.6 mm (7.8 to 12.1), respectively. Median number of screws designed in the S_1_ and S_2_ vestibules was 5 (IQR, 4 to 6) and 1 (IQR, 1 to 2), respectively. In other three cases, endoprosthesis was fixed by radial screws inserting from the prosthetic acetabulum. Median number of screws designed in the ilium was 5 (IQR, 2 to 6; Tables 2 and 3).

The anteversion and inclination of the prosthetic acetabulum were reorientated: anteversion (anatomy vs. design, 20.2° ± 2.9° vs. 23.2° ± 1.7°, *p* = 0.013) and inclination (anatomy vs. design, 41.8° ± 2.3° vs. 42.4° ± 1.7°, *p* = 0.404) as seen in Tables 2 3, and 5.

After P2/P3 resection, fixation methods were determined by the preservation of the acetabulum and ischium. In three cases, the acetabulum was partially preserved, and solid acetabulum with porous layer was applied. In 10 cases, for the acetabulum that was totally resected even with part of the pubis, pubic endoprosthesis was created. For the residual superior pubic ramus or pubic symphysis with larger intermedullary width, the pubic stem was designed, otherwise, “cap-like” endoprosthesis with a tapered inner face wrapping pubic stump was created. Different screw fixations were applied according to preservation of the narrowest zone of pubis in residual hemipelvis to be fixed. If it was preserved, screws can be inserted from the endoprosthesis acetabulum to the residual pubis; otherwise, screws would enter from the pubic endoprosthesis (Table 3).

Ischial ramus reconstruction was determined by the preservation of the ischial tubercle. If so, the endoprosthesis imitating the contralateral ischial ramus was designed. In five cases whose ischial tubercle was resected, no reconstruction was performed (Table 3).

Averaged area, length, and width of NPSPMC were 79.8 mm^2^ (40.4 to 126.2), 12.0 mm (8.6 to 16.3), and 8.3 mm (6.2 to 10.0), respectively. Median number of screws designed in the superior pubic ramus was 1 (IQR, 1 to 1). In 10 cases, whose endoprosthesis was fixed to the pubis, the average area, length, and width of RSSPMC were 200.5 mm^2^ (103.8 to 333.2), 27.4 mm (14.2 to 41.1), and 8.8 mm (5.7 to 12.4), respectively. Fixation options were stem and “cap-like” structure in eight and two cases. The stems were 20-mm long, 12-mm wide, and 6.5-mm thick. The “cap-like” structures were 5-mm deep and owned the same size with the pubic resected surface at the rim of them. Ischial screw was designed in eight cases without total resection, and number was all two (Tables 2 and 3).

All of our osteotomy guides were fixed to the tumor side of the osteotomy plane. Ten millimeters was usually set as width of osteotomy guides, and holes for inserting Kirschner's wires were distributed along the middle line of osteotomy guides.

During surgery, median number of screws inserted in the S_1_ vestibule was 4 (IQR, 3 to 4), in the S_2_ vestibule was 1 (IQR, 1 to 1), in the ilium was 3 (IQR, 1 to 5), in the pubis was 1 (IQR, 0 to 1), and in the ischium was 1 (IQR, 1 to 1). Mean intraoperative time was 292.7 min (170 to 540), and blood loss was 3538.5 ml (900 to 8200). Precise implantation was observed: anteversion (design vs. post, 23.2° ± 1.7° vs. 24° ± 3.5°, *p* = 0.380) and inclination (design vs. post, 41.9° ± 1.7° vs. 42.9° ± 2.8°, *p* = 0.333). Postoperative pathological reports showed all tumors were removed as R0 resection (Tables 4 and 5).

**Table 4:** Detailed data intra- and postoperatively

Case	S_1_ screw no. (I/D)	S_2_ screw no. (I/D)	Iliac screw no. (I/D)	Pubic screw no. (I/D)	Ischial screw no. (I/D)	Intraoperative		Postoperative AO		MSTS score							ROM of hip joint	Complications
						Time (min)	Blood loss(ml)	A (°)	I (°)	Pain	Function	Emotional acceptance	Supports	Walking ability	Gait	Total	(°)	
1	3/4	1/1	0/0	1/1	0/0	420	8200	19.5	41.4	2.0	2.0	4.0	2.0	3.0	2.0	15 (50.0%)	100.0	DWH
2	4/4	1/1	0/0	1/1	0/0	260	2500	25.8	44.0	4.0	4.0	5.0	4.0	4.0	4.0	25 (83.3%)	130.0	-
3	5/5	1/1	0/0	1/1	0/0	230	4200	20.0	40.7	3.0	4.0	4.0	4.0	4.0	4.0	23 (76.7%)	120.0	-
4	5/5	2/2	0/0	2/2	0/0	255	2100	27.7	40.9	4.0	2.0	3.0	3.0	3.0	2.0	17 (56.7%)	105.0	DWH
5	4/5	1/1	0/0	1/1	1/2	390	5600	22.9	40.2	4.0	2.0	5.0	3.0	3.0	2.0	19 (63.3%)	110.0	-
6	3/4	1/1	0/0	1/1	1/2	220	900	24.0	44.6	3.0	4.0	4.0	3.0	4.0	3.0	21 (70.0%)	130.0	-
7	3/5	1/2	1/2	0/1	1/2	270	2600	24.5	45.8	4.0	4.0	4.0	5.0	5.0	4.0	26 (86.7%)	130.0	-
8	0/0	0/0	6/6	1/1	1/2	180	2300	22.8	45.0	5.0	4.0	5.0	4.0	5.0	4.0	27 (90.0%)	125.0	-
9	0/0	0/0	3/5	1/2	1/2	170	1700	23.5	43.0	4.0	4.0	5.0	5.0	5.0	4.0	27 (90.0%)	125.0	-
10	4/5	2/2	0/0	1/1	1/2	390	5100	18.2	37.3	3.0	3.0	3.0	2.0	3.0	2.0	16 (53.3%)	110.0	-
11	0/0	0/0	5/6	2/2	0/0	210	1900	25.0	48.1	4.0	4.0	5.0	5.0	5.0	4.0	27 (90.0%)	130.0	-
12	4/5	1/1	1/2	1/1	1/2	270	2600	29.8	42.3	4.0	4.0	4.0	4.0	4.0	4.0	24 (80.0%)	125.0	-
13	4/5	1/2	0/0	1/1	2/2	540	6300	28.4	44.4	4.0	3.0	4.0	3.0	3.0	3.0	20 (66.7%)	110.0	-
Mean	-	-	-	-	-	292.7	3538.5	24.0	42.9	3.7	3.4	4.2	3.6	3.9	3.2	22.1(73.6%)	119.2	-

I/D, inserted/designed; AO, acetabular orientation; A, anteversion; I, inclination; MSTS, Musculoskeletal Tumor Society, according to Enneking et.al [19]; ROM, range of motion; DWH, delayed wound healing

**Table 5:** Comparison among anatomical, designed, and postoperative acetabular orientation

Orientation	Angles measured		*P* value
Anteversion	Anatomical	Designed	
	20.2° ± 2.9°	23.2° ± 1.7°	0.013
	Designed	Postoperative	
	23.2° ± 1.7°	24° ± 3.5°	0.380
Inclination	Anatomical	Designed	
	41.8° ± 2.3°	42.4° ± 1.7°	0.404
	Designed	Postoperative	
	42.4° ± 1.7°	42.9° ± 2.8°	0.333

**Fig. 9:**
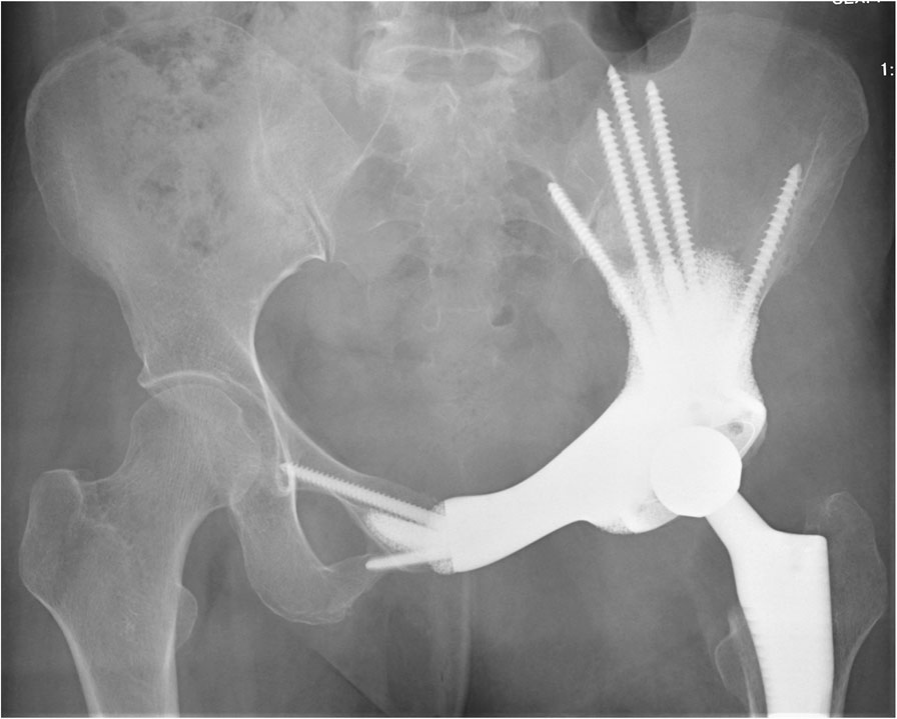
Two months after the operation, plain radiograph showed well alignment of endoprosthesis. Five screws were inserted to the ilium, and two screws were inserted to the contralateral pubis

**Fig. 10:**
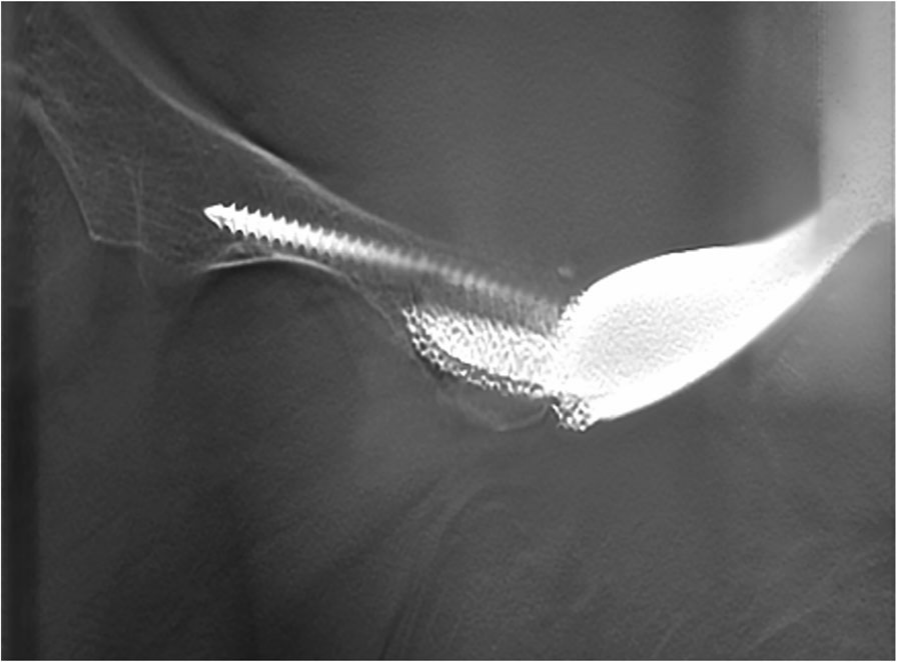
Three months after the operation, tomosynthesis-Shimadzu metal artifact reduction technology (T-SMART) showed preliminary osseointegration

Mean follow-up period of all the patients was 25.3 months (23 to 29). Averaged MSTS score was 22.1 (15 to 27) with a mean pain score of 3.7 (2.0 to 4.0), function score of 3.4 (2.0 to 4.0), emotional acceptance score of 4.2 (3.0 to 5.0), supports score of 3.6 (2.0 to 5.0), walking ability score of 3.9 (3.0 to 5.0), and gait score of 3.2 (2.0 to 4.0). Nine out of 13 require no supports, and four require a cane when walking a long distance. Mean ROM of hip joint was 119.2° (100° to 130°). No severe complications including infection, dislocation, aseptic loosening, and mechanical failure occurred (Fig. 9). T-SMART showed preliminary osseointegration three months postoperatively (Fig. 10, Table 4).

## Discussion

At present, prosthetic reconstruction is preferable for hemipelvic tumorous bone defect. A variety of prostheses have been developed since the 1970s [14], such as saddle endoprosthesis [1, 9], ice cream cone endoprosthesis [10, 11], modular hemipelvic endoprosthesis [2], and custom-made hemipelvic endoprosthesis [12, 14, 21, 22]. Despite providing acceptable clinical outcomes, endoprosthesis was associated with varies limitations. Saddle endoprosthesis and ice cream cone endoprosthesis require sufficient residual iliac bone. Besides, the saddle endoprosthesis and ice cream cone endoprosthesis cannot rebuild the intact pelvic ring, which results in the instability or looseness of the endoprosthesis. In recent years, modular hemipelvic endoprosthesis has been frequently used for different bone defects thanks to its high adaptability, nevertheless, intraoperative procedures are highly technically required to avoid erroneous implantation [23]. Custom-made hemipelvic endoprosthesis characterized by integrative design, has been reported with positive results [12, 14, 24]. No matter, manufactured by computerized numerical control technique, rapid casting technique, or 3D printing technology, these prostheses have a common shortcoming: absence of porous structure, resulting in poor osseointegration and inevitable mechanical failure [12, 14, 22]. Therefore, improving design and fabricating technique of custom-made endoprosthesis seems crucial.

With the development of 3D printing technology and profound understanding in osseointegration, novel 3D-printed custom-made endoprosthesis with porous structure has been reported with good early results [15, 23, 25–27]. However, the details of endoprosthesis design was still unclear [27]. In our study, we firstly described the detailed design methodology step by step.

First of all, the feature of the preliminarily designed endoprosthesis needs modification. Simplification such as minimizing iliac wing and removing ischial spine and posterior iliac spines are good for wound healing, nerve protection and convenient implantation. Holes along endoprosthesis crest are for muscle reattaching, such as rectus femoris which is important for hip function. Again, the main body should be divided into solid and porous structures in order to reduce endoprosthesis weight, guarantee endoprosthesis strength, and enhance bone ingrowth [28]. Previous studies have reported that porous structure with pore size of 300 to 800 μm and porosity of 70% can enhance bone ingrowth [29–33]. And post-processing procedure seems challenging in the porous structure under 600 μm-pore size [34]. Consequently, the porous structure with 600 μm pore size and 70% porosity was applied, and the follow-up outcomes showed well osseointegration in our patients. To achieve equal weight between resected specimen and endoprosthesis, the VPPS was modulated. Weight of resected specimen was calculated as product of its density and volume, and weight of endoprosthesis was calculated as total weight of porous and solid structures. The resected specimen is mixed by tumor, muscle, trabecular bone, and cortical bone so that its density was defined as the density of trabecular bone for tough estimation. Therefore, in our series, the VPPS was calculated as between 53 and 86%.

Integral configuration design was done, and then regional design was performed. Design after P1 resection plays the most important role [2, 16]. The major challenge is to fix the endoprosthesis rigidly. Long-term stability can be achieved by a well-designed porous structure, and initial stability is regarded as the fundamental factor. Firstly, flat connecting surface is required for tight connection and smooth intraoperative procedure. Secondly, all screws did not go through the sacroiliac joint, and the distribution of screws was determined by the residual volume of the ilium. Additionally, more and longer screws fixed in the S_1_ vestibule were designed during preoperative simulation, and a back-up screw was designed in case of an unexpected situation [35]. Moreover, in the iliac screw design, radial distribution of screws was good for stress dispersion. As a result, in our follow-up, no mechanical failure was observed.

Design after P2 resection concentrates on the location and orientation of the prosthetic acetabulum which is crucial for hip joint function. The contralateral acetabulum was used for location imitation [15, 26]. Meanwhile, soft tissue removal and acetabular dysplasia correction were taken into consideration for acetabular orientation. Finally, thanks to our precise implantation, there were no significant differences between designed and postoperative acetabular location and orientation. And no dislocation was encountered.

In addition, reconstruction after P3 resection was performed to rebuild intact pelvic ring. Despite the extended plate being used assistantly for pubic endoprosthesis fixation in a previous study [21], it required more exposure as well as offered eccentric and poor force conduction. Therefore, central fixation of pubic endoprosthesis with specially designed connecting parts including stem or “cap-like” structure was designed basically according to the size of RSSPMC. Pubic stem is usually used in relatively larger pubis whose width of the resected surface is over 7.5 mm. But the “cap-like” structure is usually applied in thinner pubis whose width of resected surface is within 7.5 mm. In consideration of separating tendency after the destruction of pelvic ring integrity, assistant screws inserting to the residual pubic ramus are needed. The curvature of the superior pubic ramus reduces screw length in the bone; therefore, pubic screw is regulated by inserting from the acetabulum or pubic endoprosthesis for better fixation. With regard to ischial reconstruction, reports are quite limited. Our ischial design balances implantation difficulty and load transmission. Due to the deep location of the ischium, a back-up screw was applied to avoid fixation failure.

Intraoperatively precise osteotomy, proper endoprosthesis implantation, and correct screw insertion are critical in the operation of 3D-printed prosthetic reconstruction. Osteotomy guides were applied for providing desired osteotomy plane with less exposure and instrument requirement during operation [25, 26]. Additionally, due to the complex anatomy of the pelvis and severe displacement of the residual pelvic bone after resection, proper placement of endoprosthesis is technically demanding: (1) plastic endoprosthesis model can be used for the assessment of precise osteotomy; (2) conformation of well placement should be done before inserting screws by checking the acetabular orientation, pelvic continuity near osteotomy plane; (3) fixation between the sacrum or ilium and endoprosthesis is prior, then the reduction and fixation between superior pubic ramus and endoprosthesis; and (4) reduction using lag screw is required due to approachability of the ischium.

We also recognized limitations in this study. First of all, our short follow-up period might limit observation of unknown drawbacks. However, stable fixation, well osseointegration, and good functional outcome have been observed. Thus, long-term outcomes can be expected. Secondly, single uniform porous structure was applied. A porous structure with gradient porosity imitating the cortex bone and trabecular bone might be the best option for osseointegration; however, it is still challenging due to not necessarily uniform shrinkage. In addition, biomechanical analysis was not included in this study; therefore, finite element analysis and vitro tests focusing on (1) different porous structures, varying in pore size, porosity, and geometry configuration; (2) different VPPS; (3) different distributions of porous and solid structures; (4) different diameter, distribution and orientation of screws; (5) different fixation methods such as plate assisting fixation and lag screw fixation; and (6) different two pubic fixation methods in our design will be conducted to estimate the biomechanical performance and improve our endoprosthesis. Last but not least, although our series is one of the largest studies in 3D-printed custom-made hemipelvic endoprosthesis replacement, sample size with thirteen patients is still small which limited the power of the series. Therefore, a larger multi-institutional study is needed to ideally compare this approach with other types of reconstruction.

## Conclusions

This study firstly presents detailed design methodology and related surgical techniques of 3D-printed custom-made hemipelvic endoprosthesis in treating malignancies involving acetabulum. Reconstruction with this kind of patient-specific implant is a multi-step process that involves measurement, design, manufacture, and surgery. Precise resection is performed with osteotomy guides and provided with foundation for complete reduction. Expectable bone ingrowth is conducted by highly matching surface, optimal porous structure, and well initial stability which is achieved by precise matching in 3D space including anatomical-conforming shape and proper fixation methods. Therefore, long-term outcome can be promising. Despite favorable outcomes, we observed some imperfections in the preoperative design and surgical application. More works are required in further study.

## Data Availability

The data and materials are available from the medical records department of West China Hospital. The datasets used and analyzed during the current study are available from the corresponding author on reasonable request.

## Abbreviations

3D: Three dimensional
S1: First sacral
S2: Second sacral
NPSPMC: The narrowest zone of superior pubic medullary cavity
RSSPMC: The resected surface of superior pubic medullary cavity
IQR: Interquartile range
PR: Plain radiograph
CT: Computerized tomography
MRI: Magnetic resonance imaging
SPECT: Single-photon emission computed tomography
PET/CT: Positron emission tomography/ computerized tomography
T-SMART: Tomosynthesis-Shimadzu metal artifact reduction technology
MSTS: Musculoskeletal Tumor Society
ROM: Range of motion
VPPS: Volume proportion of the porous structure
*ρ*_*t*min_: Minimum density of pelvic trabecular bone
*ρ*_tmax_: Maximum density of pelvic trabecular bone
*ρ*_Ti_: Density of titanium alloy
*ρ*_P_: Mean density of porous structure
*V*_e_: Volume of total endoprosthesis
*V*_r_: Volume of resected specimen
